# Association between serum Vitamin D deficiency and Knee Osteoarthritis

**DOI:** 10.31138/mjr.30.4.216

**Published:** 2019-12-31

**Authors:** Hasan Anari, Afsaneh Enteshari-Moghaddam, Yeghaneh Abdolzadeh

**Affiliations:** 1 Department of Radiology, School of Medicine, Ardabil University of Medical Sciences, Ardabil, Iran,; 2 Department of Internal Medicine, School of Medicine, Ardabil University of Medical Sciences, Ardabil, Iran,; 3 School of Medicine, Ardabil University of Medical Sciences, Ardabil, Iran

**Keywords:** vitamin D, osteoarthritis, knee, radiography

## Abstract

**Objective:**

Levels of Vitamin D may influence the development of knee osteoarthritis (OA), which is one of the most common joint diseases. The aim of this study was to investigate the association between serum Vitamin D deficiency and knee OA in Ardabil and Iran.

**Methods:**

One hundred fifty-eight consecutive patients referred to rheumatology clinic of Ardabil City Hospital were recruited in the study. All the participants underwent x-rays in two anterior-posterior and side views of the knees. Staging of knee OA was done according to Kellgren-Lawrence criteria. Seventy-nine individuals with clinical and radiographic signs of knee OA were defined as the case group, and those without clinical and radiographic signs of the disease were defined as a control group. Haematology and biochemical profile including measurement of 25-hydroxyvitamin D serum level was performed in the participants.

**Results:**

The mean age of patients and controls were 54.12 ± 4.67 and 55.37 ± 5.12 years, respectively. The average serum vitamin D in OA patients and controls were 26.8±6.2 ng/ml and 28.1±5.3ng/ml, respectively (p=0.36). There was a significant association between serum vitamin D and staging of knee OA (p=0.001). Based on vitamin D levels, most of patients with vitamin D deficiency were in stages III and IV.

**Conclusion:**

The findings of the study suggest that vitamin D deficiency should be considered in patients with OA and treated accordingly.

## INTRODUCTION

Vitamin D deficiency is common, with several epidemiologic studies having demonstrated that low levels of vitamin D are associated with higher levels of knee pain, increased prevalence of osteoarthritis (OA), as well as development and progression of knee OA.^1–4^

Vitamin D is a steroid and fat-soluble substance with sunlight helping in its construction. Fish, milk, butter, egg yolks and mushrooms are good sources of vitamin D. Vitamin D metabolites are actually hormones and hormonal precursors, which could be made in vivo under appropriate biological conditions, and converts to calcitriol during various metabolic changes.^5^ The main function of vitamin D is to absorb calcium and phosphate: vitamin D deficiency in children leads to rickets, and, in adults, to osteomalacia.^3,6–9^

In men with vitamin D deficiency, the prevalence of OA has been reported to be significantly higher compared to those with sufficient levels.^10^ Other studies showed that vitamin D deficiency is age-dependent and could be high in elderly patients; these patients are at higher risk of OA in their life.^11–12^

Mild to moderate vitamin D deficiency could be asymptomatic, whereas long-term vitamin D deficiency can cause hypocalcaemia with secondary hyperparathyroidism and bone mineralization disorder.^6,13– 15^

OA is the most common form of arthritis. Although OA can affect both spine and peripheral joints, it most commonly occurs in the joints of the hands, neck, waist, knees and pelvis. Existing treatments for OA could reduce disease progression and pain in patients, and improve their joint functions.^14–17^

Many factors such as age, gender, bone deformities or abnormalities, weight, profession and also many underlying diseases such as diabetes, hypothyroidism, gout or Paget’s bone disease could increase the risk of OA.^18–20^

Given the association between OA and vitamin D deficiency, the aim of this study was to investigate the association between serum vitamin D deficiency and knee OA in Ardabil, Iran.

## METHODS AND MATERIALS

### Study design and patients

This was a case-control study, and a total of 158 patients with knee pain referred to rheumatology clinic of Ardabil City Hospital were studied. The sampling method was census, 79 individuals with clinical and radiographic signs of knee OA were defined as a case group, and those without clinical and radiographic signs of knee OA were defined as a control group. The controls were matched for age and gender. Patients with OA who underwent surgery or had history of trauma and inflammatory arthropathy were excluded from the study.

### Sampling and data collection

Demographic information such as age, gender, height, weight, place of residence, history of smoking, education level, history of arthritis, etc. were collected from the participants. Radiographs were done in two posterior anterior and lateral views of the knees with radiographic apparatus (Shimadzu Diagnostic Radiography RAD speed series D digital).

The Kellgren-Lawrence grading system is a radiological classification of knee OA. It ranges from grade 0 to grade 4, and is based on x-ray findings. Radiographs were reviewed by a radiologist and divided into stages as below:

- **Grade 1:** doubtful narrowing of joint space and possible osteophytic lipping
- **Grade 2:** definite osteophytes, definite narrowing of joint space
- **Grade 3:** moderate multiple osteophytes, definite narrowing of joints space, some sclerosis and possible deformity of bone contour
- **Grade 4:** large osteophytes, marked narrowing of joint space, severe sclerosis and definite deformity of bone contour

Venous blood samples were obtained from both groups and serum 25-hydroxyvitamin D levels were also measured in the laboratory by radioimmunoassay. Serum 25-hydroxy vitamin D level 30 ng/ml and above was defined as normal, 20–29 ng/ml was considered scant, and 20 ng/ml and lower indicated deficiency.

### Statistical analysis

Collected data through checklists were entered into SPSS version 22 and analysed using descriptive statistics in the form of tables and graphs and analytical statistics such as Kruskal-Wallis test to compare means between two groups and chi-square test to determine the relation between qualitative variables. P<0.05 was considered as significant.

### Ethical approval

Consent was obtained from all patients. The dissertation was registered in the Ethics Committee of Ardabil University of Medical Sciences with code IR.ARUMS. REC.1397.052.

## RESULTS

The mean age of patients and controls was 54.1±4.7 and 55.4±5.2 years, respectively (p=0.45). Most persons in both groups were in the age group between 50–60 years. The average height of patients and controls were 168.7±5.2 cm and 167.4±4.9 cm, respectively. The mean weight in patients and controls were 81.4±6.4 and 80±5.6 kg, respectively. The mean BMI in patients and controls were 28.7±2.9 and 28.6±2.7, respectively. There was no significant difference in height, weight and BMI between the two groups. Most of the subjects in the case and control groups were housewives (*Table 1*). The average serum vitamin D in OA patients and controls were 26.8±6.2 ng/ml and 28.1±5.3 ng/ml, respectively, and the difference was not significant based on Kruskal-Wallis test (*Table 2*). The rate of smoking use in patients and controls were 13.9% and 10.1%, respectively. In patients with knee OA according to Kellgren-Lawrence criteria, 16 (20.2%) patients were in grade 1, 35 (44.3%) in grade 2, 20 (25.3%) in grade 3 and 8 (10.2%) in grade. There was a significant association between serum vitamin D and staging of knee OA (p=0.001). Based on vitamin D levels, most patients with vitamin D levels below 20 were in grades 3 and 4. (*Table 3*)

**Table 1.** Baseline characteristics of the patients and controls.

**Characteristics**		**Case**		**Control**		**p-value**
		**n**	**%**	**n**	**%**	
Gender	Female	31		33	41.8	0.48
	Male	48		46	58.2		
Age (Mean±SD)		54.1±4.7		55.4±5.2		0.45	
Weight (Mean±SD)		81.4±6.4		80±5.6		0.54
BMI (Mean±SD)		28.7±2.9		28.6±2.7		0.6
Profession	Housekeeper	35	44.3	39	49.5	0.52
	Unemployed	29	36.8	28	35.4	
	Employed	15	18.9	12	15.1	
Residence	Urban	54	68.3	57	72.1	0.39
	Rural	25	31.7	22	27.9	
Education	Illiterate	33	41.7	34	43.2	0.26
	Diploma and lower	35	44.2	35	44.2	
	Graduate	11	13.9	10	12.6	

**Table 2.** Distribution of Vitamin D level in patients in two groups.

**Vitamin D**		**Case**	**Control**	**p-value**
**Mean±SD**		26.8±6.2	28.1±5.3	0.36
**Level**	< 20 ng/ml	22 (27.8%)	19 (24%)	0.36
	20–30 ng/ml	37 (46.8%)	39 (49.3%)	
	>30 ng/ml	20 (25.4%)	21 (26.8%)	

**Table 3.** Association between stage of disease and Vitamin D level.

Grading Level vitamin D	**1**		**2**		**3**		**4**		**p-value**
	**n**		**n**	**%**	**n**		**n**	**%**	
< 20 ng/ml	2	12.6	3	8.6	11	55	6	75	0.001
20–30 ng/ml	7	43.7	21	60	7	35	2	25	
>30 ng/ml	7	43.7	11	31.4	2	10	0	0	
Total	16	20.2	35	44.3	20	25.3	8	10.2	

In subgroup analysis, the mean 25-OHD in OA patients aged <60 years was similar to patients aged >60 years (27.8±5.7 vs. 25.2±5.8 ng/ml, p=0.08).

## DISCUSSION

The main finding of this study is the association between the grade of knee OA and vitamin D levels. Our observations concur with those of Bergink et al., who showed that low vitamin D intake increases the risk of radiographic progression of knee OA, especially when the patient’s bone mineral density (BMD) is low. Therefore, improving vitamin D levels in patients may have a protective role against the development of OA, especially in those patients with low BMD.^4^ Similarly, McAlindon et al. demonstrated significant relationship between serum vitamin D level and progression of OA, so that those with more advanced OA had lower vitamin D levels.^3^ In contrast, Al-Jarallah et al. did not establish any relationship between vitamin D levels and radiographic grade of knee OA.^21^

In a study by Muraki et al., no significant relationship was found between vitamin D levels and radiographic OA of the knee, but low levels of 25-OHD were more associated with knee pain.^16^ This study is not in line with our study, because in contrast to the above study, there was a significant association between vitamin D levels and knee OA. The average serum levels of vitamin D in cases was 26.78±6.15 ng/ml and in controls was 28.11±5.33 ng/ml; the difference between the two groups was not significant. In patients with knee OA, according to Kellgren-Lawrence criteria, 16 (20.2%) patients were in grade 1, 35 (44.3%) in grade 2, 20 (25.3%) in grade 3 and 8 (10.2%) in grade 4. Based on vitamin D levels, most patients with vitamin D levels below 20 were in grades 3 and 4. There was a significant correlation between patients according to Kellgren-Lawrence criterion in terms of serum vitamin D level (P= 0.001).

In our study, we did not find any correlation between grade progression of knee OA and demographic parameters such as gender, occupation and educational level of patients. A study by Felson et al. showed that those with lower levels of vitamin D were more likely to have knee OA, but this was not correlated with the severity of symptoms.^22^

In the current study, we confirmed previous reports regarding the lack of significant difference in vitamin D levels between OA patients and controls.^2,4,22–23^ In addition, our study is in agreement with Heidari et al., who did not describe any significant difference of vitamin D between age groups above and below 60 years.^1^

In our study, we found significant relation between knee OA grading and vitamin D level: results showed that most patients with vitamin D deficiency were in grade three and above, which could be a late phase in learned pain. Results also showed that patients with normal vitamin D were in grade 2 and below. This could mean that low levels of vitamin D may be play a critical role in OA progression and its pathogenesis. Similar to our study, Murat and Laslett in their studies showed the significant relation between vitamin D levels and OA occurrence.^16,24^ Also, in the Cakar et al. study, this relation was not significant, which is not in line with our study results, since in the above study, most patients were in grade 2.^25^

## CONCLUSION

Results showed that there was a significant association between knee OA grade based on Kellgren-Lawrence criteria and serum vitamin D levels. According to the importance of the subject, further clinical and interventional studies are needed in this area.
